# Harnessing Polymeric Xerogels for Enhanced Wound Care: Properties, Mechanisms, and Applications

**DOI:** 10.1021/acsmaterialsau.5c00134

**Published:** 2025-11-27

**Authors:** Amrita Kumari, Sweta Acharya, Gautam Singhvi, Ashwin Mali, Ankit Jain

**Affiliations:** † Department of Pharmacy, Armed Forces Medical College, Solapur Road, Pune, Maharashtra 411040, India; ‡ Department of Pharmacy, Birla Institute of Technology and Science-Pilani, Pilani Campus, Pilani, Rajasthan 333031, India; § Poona College of Pharmacy, Bharati Vidyapeeth Deemed University, Erandwane, Pune, Maharashtra 411001, India

**Keywords:** xerogels, wound healing, controlled drug release, polymers, nanotechnology, biocompatibility, tissue regeneration, scaffolds, hemostasis

## Abstract

Wounds significantly impact an individual’s quality of life, necessitating a tailored approach to treatment based on the wound’s stage of healing and condition. Exudate plays a natural role in recovery, but excessive amounts can complicate wound management, creating a need for advanced therapeutic solutions. Consequently, there is an ongoing demand for advanced therapeutic solutions and innovative wound care devices. Xerogels are gaining recognition as promising materials in wound healing therapeutics due to their unique properties and multifunctional applications. These nanoporous materials, characterized by their large surface area and biocompatibility, can be engineered using various polymers to enhance their effectiveness for specific wound care applications. Their ability to support clot formation and promote tissue regeneration makes them particularly valuable for addressing exudative and chronic wounds. This review offers an in-depth examination of emerging research on xerogels in wound treatment, assessing the current landscape and identifying potential applications of xerogels in various forms including films, grafts, scaffolds, and particles. Additionally, we explore various mechanisms of polymer-based xerogel function and summarize recent patents related to this innovative technology. As research in this area progresses, xerogels utilizing different polymers offer advanced solutions for future wound care therapies.

## Graphical abstract

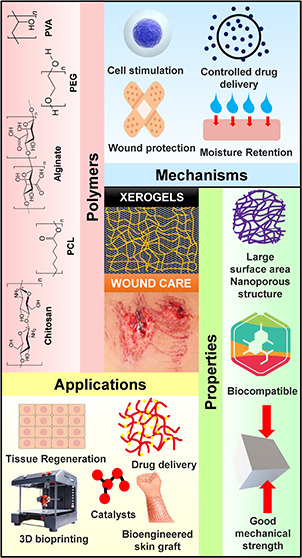

## 1 Introduction

A wound alters the skin’s and deeper tissues’ typical configuration and functionality due to external injury, surgical procedures, or existing medical conditions.^1,2^ Wounds can vary in severity and type and can be categorized as acute or chronic wounds based on their cause, depth, and healing time.^3^ While acute wounds heal promptly and predictably, chronic ones take much longer.^4^ Acute wounds are typically caused by surgical incisions or external injuries such as cuts, abrasions, punctures, or burns. In contrast, chronic wounds are injuries to the deeper layers of the skin. These wounds persist because of underlying factors such as diabetes, inadequate blood circulation, extended pressure, or weakened immune systems.^5^ Typical chronic wounds include diabetic foot ulcers, pressure ulcers (also known as bedsores), venous and arterial insufficiency ulcers. Wound healing represents one of the most complex and fascinating biological processes, involving an intricate cascade of cellular and molecular events that restores tissue integrity following injury. The healing of acute wounds progresses through several organized phases: hemostasis, inflammation, proliferation, and remodeling. Hence, proper care and management are essential to ensure that acute wounds heal effectively and without complications. In contrast, chronic wounds often require specialized and ongoing care to manage infection, reduce pain, and promote healing.^6^

Even with considerable progress in medical science, wound healing remains challenging, particularly for chronic wounds that fail to progress through the typical stages of healing. Traditional wound management approaches, while foundational, often fall short in addressing the microenvironmental requirements necessary for optimal healing. Conventional wound treatment methods such as standard dressings—including gauze, hydrocolloids, and foam, are widely adopted due to their affordability, ease of use, and accessibility. These dressings offer basic wound protection, absorb exudates, and help maintain a moist healing environment. Similarly, topical ointments and creams, such as antibiotic formulations (e.g., Neosporin), silver sulfadiazine, and herbal preparations, are commonly used for infection control and epithelialization, requiring minimal clinical supervision and offering cost-effective care. However, these conventional approaches are generally limited in their ability to provide controlled drug release, targeted delivery, or bioactive modulation of the wound microenvironment. They often necessitate frequent dressing changes and may be inadequate in preventing biofilm formation and managing chronic inflammation. Recent developments in wound healing techniques have focused on enhancing the body’s innate healing mechanisms and addressing the limitations of conventional therapies. Several groundbreaking approaches have emerged in wound healing management, including nanotherapeutics, which typically employ the use of nanoparticles for direct delivery of drugs to the wound site, enhancing treatments’ effectiveness and reducing untoward effects;^7^ stem cell therapy utilizes stem cells to promote tissue regeneration and accelerate healing, particularly in chronic wounds.^8^ In the case of bioengineered skin grafts, artificial skin substitutes mimic the properties of natural skin and provide a scaffold for new tissue growth.^9^ 3D bioprinting creates customized wound dressings and skin grafts using 3D printing methodology, which uses cells and bioinks for extreme control over the structure and composition of the materials.^7^

In some cases, the growth factors have been incorporated into wound dressings to stimulate cell proliferation and tissue repair.^8^ There have also been innovations in wound dressings that sustain a moist environment, offer an antimicrobial defense, and accelerate healing.^9^ Advancements in wound care strategies are illustrated in Figure 1.

**Figure 1:**
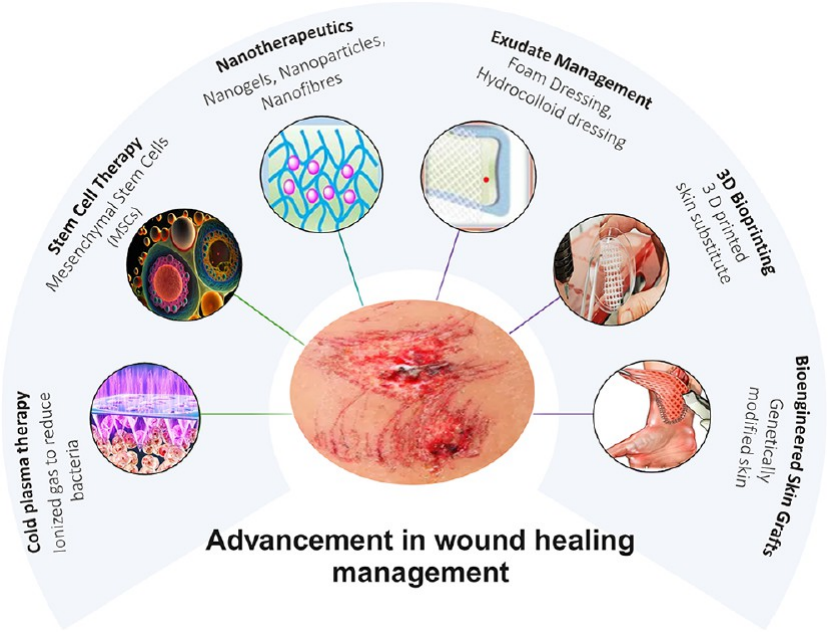
Advancements in wound healing management.

By harnessing the latest breakthroughs in biotechnology and materials science, researchers and clinicians create more efficient and personalized treatments that enhance healing outcomes and improve patients’ quality of life. One recent advancement in wound healing technology is Xerogels. Xerogels are a type of porous material formed by the drying of gels, where the liquid component is removed through evaporation at ambient pressure.^10^ This distinctive formation mechanism endows xerogels with exceptional properties, including high porosity, large surface area, tunable pore architecture, and the ability to incorporate diverse therapeutic agents during synthesis. The progress in xerogel development has advanced considerably over the years, fueled by breakthroughs in materials science and the demand for efficient, affordable solutions across various applications. Unlike aerogels, which require supercritical drying, xerogels are produced through a more straightforward and cost-effective process, making them more accessible for practical use.^11^ The term “xerogel” was initially introduced by Freundlich to describe gels that undergo shrinkage or swelling upon drying. According to the International Union of Pure and Applied Chemistry (IUPAC), a xerogel is defined as an “open network formed by removing all swelling agents from a gel.” Initial studies focused on understanding the drying process and the structural changes in the gels. Researchers explored different drying techniques to minimize shrinkage and cracking, common issues in xerogel production.^12^ The development of sol–gel processes enables better control over the properties of xerogels, resulting in materials with a high surface area and porosity. Xerogels can be synthesized from various organic and inorganic precursors, including biopolymers and nanocellulose.^13^ Their unique structure allows them to be used in multiple fields, such as drug delivery, environmental remediation, and as catalysts or adsorbents.^14^ Controlling their morphology and properties during synthesis further enhances their versatility and functionality.^15^

Xerogel-based therapeutic strategies encompass a broad spectrum of innovative approaches, from simple drug-loaded matrices to complex stimuli-responsive systems capable of real-time adaptation to wound conditions. These platforms can be designed to deliver antimicrobial agents for infection control, growth factors for enhanced cellular proliferation, anti-inflammatory compounds for inflammation modulation, and various other bioactive molecules targeting specific aspects of the healing process.^16^ The temporal control of therapeutic release can be precisely tuned through manipulation of xerogel composition, cross-linking density, and pore structure, enabling sustained and localized drug delivery that matches the kinetics of the wound healing phase.^17^

Numerous research papers have investigated xerogels’ fabrication, characterization, and diverse biomedical applications. While Yahya et al. examined the wound-healing properties of antibacterial cellulose-based aerogels^18^ and Bernardes et al. focused on aerogels in wound management,^19^ there is a notable lack of comprehensive reviews explicitly addressing the application of xerogels in wound care. This review aims to accentuate the significant role of xerogels in wound management. It encompasses all aspects of the xerogel, including its evolution, fabrication techniques, factors, and mechanisms. We also compared xerogels and aerogels, highlighting their distinct properties and advantages in wound healing. Additionally, it explores various patents related to xerogels, showcasing innovative applications in wound care settings. This review will further summarize the contribution of xerogels in managing various types of wounds, thereby enhancing our understanding of their application in wound care management and filling a crucial gap in the current research.

## 2 Wound Pathophysiology and Its Caring Needs

Wound healing is a sophisticated biological process involving complex cellular and molecular interactions. Wounds are classified as acute or chronic based on their healing trajectory, with acute wounds resulting from sudden trauma or surgical intervention that typically heal within 2–4 weeks through predictable repair mechanisms, while chronic wounds represent pathological deviations from normal healing, failing to progress through orderly phases within 3 months due to underlying conditions, such as venous insufficiency, diabetes, or arterial disease. Wound healing typically progresses through four distinct stages: hemostasis, inflammation, proliferation, and remodeling. Each phase is essential in repairing damaged tissue and restoring its structural integrity and function.^2,20^

### 2.1 Hemostasis

Hemostasis is the immediate response to a vascular injury and is crucial for preventing blood loss. This phase consists of three sequential steps. The first step is vasoconstriction, in which the blood vessels constrict to reduce the blood flow. It follows primary hemostasis, where the platelets adhere to the exposed collagen in the subendothelial matrix and aggregate to form a temporary platelet plug. Lastly, there is secondary hemostasis in which the coagulation cascade is activated, resulting in the conversion of soluble fibrinogen into insoluble fibrin strands that stabilize the platelet plug, forming a thrombus that not only halts bleeding but also releases growth factors essential for subsequent healing phases.^21^

### 2.2 Inflammation

Following the hemostasis phase, the inflammatory phase begins, lasting a few days to a week. It begins with neutrophils, the first responders to the injury, which work to eliminate bacteria and clear debris from the wound site. Shortly after, monocytes arrive and differentiate into macrophages, which play a vital role in cleaning the wound and phagocytosing damaged cells and pathogens. They also release cytokines and growth factors that signal other cells to participate in the healing process. During this phase, several essential processes begin, such as angiogenesis, which forms new blood vessels; fibroplasia, which generates granulation tissue; and re-epithelialization, which restores the skin’s protective barrier. These actions typically commence within 48 h after the injury and are essential for setting the stage for the proliferative phase of healing. Through these efforts, the inflammatory phase helps control infection and lays the groundwork for tissue regeneration.^22^

### 2.3 Proliferation

After the inflammatory phase is resolved, the body enters the proliferative phase, which focuses on tissue restoration. This phase typically lasts for several weeks and involves several critical processes. First, angiogenesis occurs as endothelial cells proliferate and migrate to form new capillaries, ensuring adequate oxygen and nutrient supply to the healing tissue. Concurrently, fibroplasia occurs, with fibroblasts synthesizing collagen and other extracellular matrix (ECM) components, resulting in the formation of granulation tissue that provides structural support. Additionally, re-epithelialization involves keratinocytes migrating across the wound bed to cover it with new epithelial cells, which restores the skin barrier. This phase is essential for rebuilding and repairing the damaged tissue, paving the way for the subsequent maturation phase of wound healing.^22^

### 2.4 Remodeling

The last phase of wound healing follows the remodeling phase, which involves the maturation of the scar tissue. During this period, the scar strengthens and becomes less vascularized, achieving a more normal appearance and functionality. Maintaining a balance between collagen synthesis and degradation is crucial, as it prevents the scar from becoming overly prominent. Eventually, the remodeled tissue seeks to regain its preinjury elasticity and strength, although it may not fully resemble the original skin structure. This maturation process can take several months to years. More profound injuries may result in noticeable scarring, while minor superficial wounds may heal with minimal marks, showcasing the skin’s adaptability. This phase is vital for restoring aesthetics and functional integrity, leading to optimal outcomes in wound healing, as illustrated in Figure 2.^23^ Chronic wounds exhibit persistent inflammation, excessive matrix metalloproteinase activity, prolonged polymorphonuclear neutrophil activation, bacterial colonization, and impaired growth factor signaling, creating a self-perpetuating cycle of tissue destruction that requires targeted therapeutic interventions addressing underlying pathophysiology to restore normal healing cascades and achieve optimal functional outcomes.^20,24^

**Figure 2:**
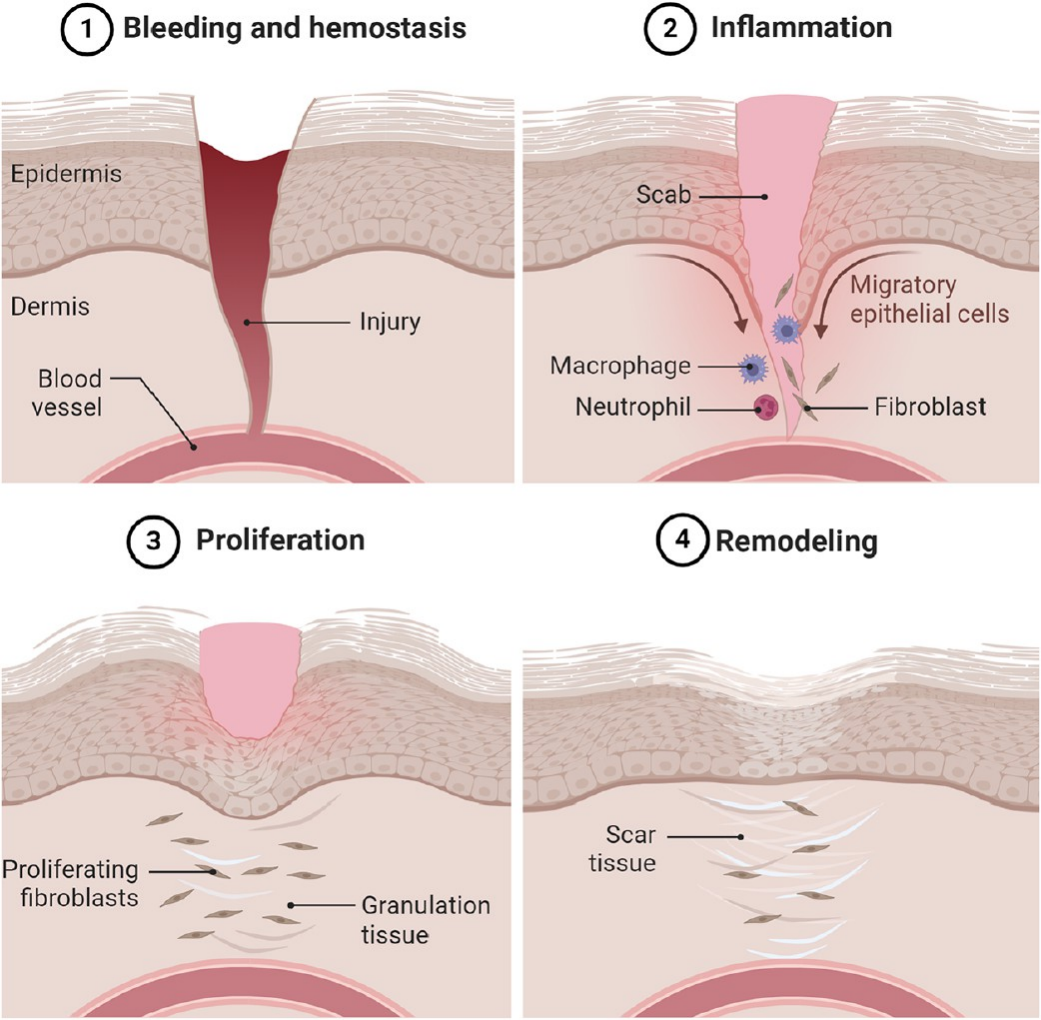
Schematic showing the phases of wound healing.

## 3 Xerogels

### 3.1 Evolution of Xerogels

The development of xerogels is intrinsically linked to the pioneering work of Samuel Kistler in the early 1930s, when he first introduced the concept of aerogels by using supercritical drying conditions to remove the liquid from a wet gel; his work was published in the journal Nature in the year 1931.^25^ While Kistler’s initial focus was on aerogel, his foundational work established the principles that later led to the development of xerogel. The process of sol–gel synthesis was first reported way back in 1845 by Ebelman, followed by Geffcen and Berger who established the process of achieving the sol–gel technique in the 1930s.^26^ The evolution of the sol–gel technique became fundamental to the production of xerogels. The xerogel as a separate material for biomedical application came into existence in late 1969, when a rectal dosage form of xerogel was prepared and compared with classical suppositories.^27^ The first xerogel used for wound healing originated in the mid 1970. The Debrisan, also known as Dextranomer, manufactured by Pharmacia GB, is a dry, spherical bead composed of dextran that has been cross-linked with epichlorohydrin and sodium hydroxide. It was the first marketed product of xerogel in the field of wound healing, introduced in Sweden around 1975 based on clinical trials as a wound cleansing agent and for the management of superficial ulceration. Since then, several advancements in xerogel dressing have been made, either to be used as a scaffold, electrospun fiber, or as a smart dressing that releases the drug based on stimuli such as a change in pH, temperature, etc.^28–30^

### 3.2 Comparison of Xerogels with Aerogels and Hydrogels

Xerogels are solid materials derived from gels after removing their liquid content through processes, such as evaporation or freeze-drying. During the drying process, the liquid is extracted from the gel while its original shape and structure are preserved as much as possible. Xerogels and aerogels are both highly porous materials with unique properties. However, they differ significantly in their structure, porosity, and production methods, as summarized in Table 1.^11,12,31–34^ Both xerogels and aerogels have unique advantages, making them suitable for different applications, as shown in Figure 3. However, there are some advantages of xerogels over aerogels, which lie in their cost-effectiveness during production, as they are produced through ambient pressure drying, which is less energy-intensive than the supercritical drying process used for aerogels.^13^ In addition to that xerogels possess greater mechanical strength and durability than aerogels, which are ultralight and fragile.^10^ The lower porosity of Xerogels is ideal for sustained drug delivery, as it slows drug release. In contrast, the higher porosity of aerogels leads to faster release, which is often unsuitable for conditions like cancer treatment.^13^ These advantages make xerogels a practical choice for various industrial and biomedical applications where cost, mechanical strength, and ease of handling are critical factors.^33^

**Figure 3:**
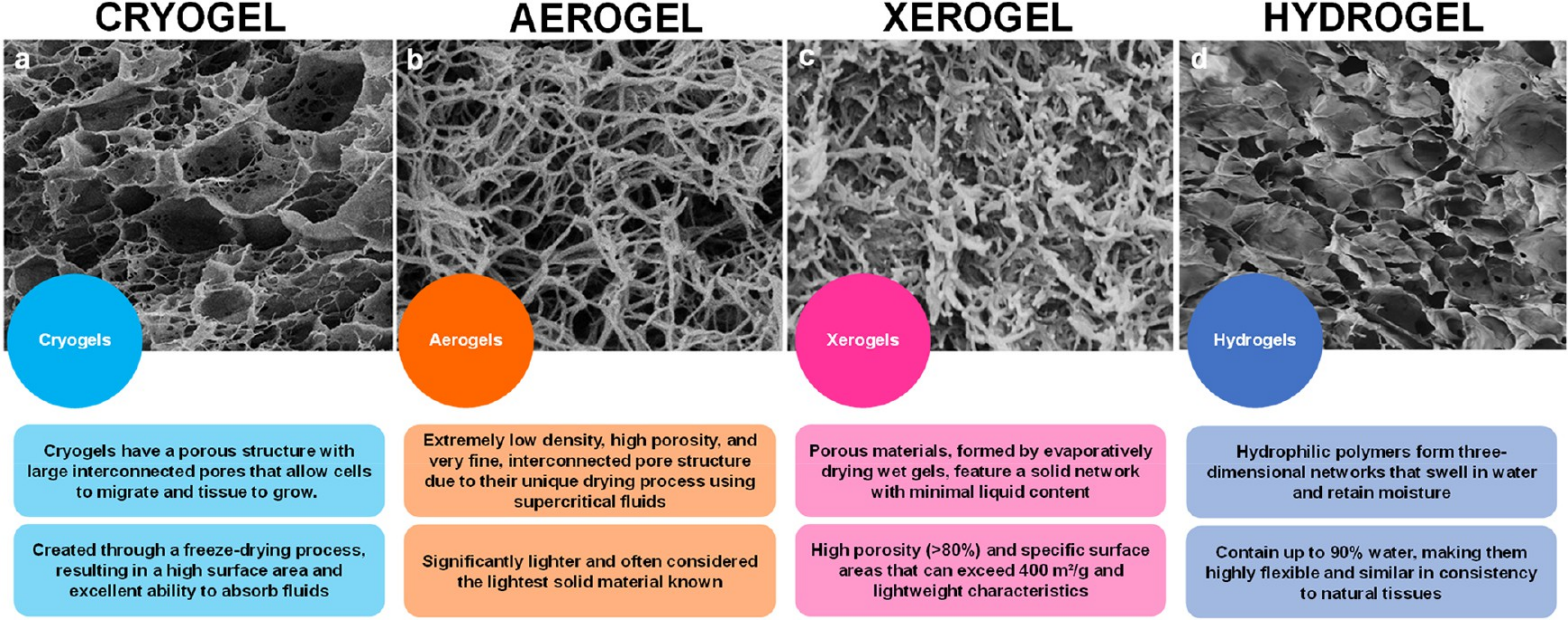
Schematic diagram representing various types of gels and their properties, (a) Cryogel, (b) aerogels, (c) xerogels, adapted with permission from^36^ under © 2021 Elsevier Ltd. All rights reserved, and (d) hydrogels, Reprinted with permission under a Creative Commons CC BY 4.0 license from.^37^ Copyright 2023, MDPI, Basel, Switzerland.

**Table 1:** Comparative Overview of Xerogels and Aerogels

Characteristics	Aerogel	Xerogel
Production	Aerogels are created by removing the liquid content of a gel with a supercritical fluid. This process involves high pressure and temperature, allowing the liquid to be extracted without collapsing the gel structure, and hence, the liquid is replaced with air.	Xerogels are formed by evaporating the liquid component of a gel at room temperature or using an oven dryer. This slower drying process can lead to shrinkage and cracking.
Density	Extremely low density, making them some of the lightest solid materials	Higher density compared to aerogels
Porosity	High porosity, often between 90 and 99.8%, resulting in a large surface area	Lower porosity than aerogels, but still significant
Shrinkage	Minimal shrinkage during the drying process, maintaining their original	Higher shrinkage during the drying process can affect their structural integrity.
Application	Mainly used as a thermal insulator	Versatile in applications like drug delivery and catalysis

Hydrogel, in comparison to xerogels, has a three-dimensional network of hydrophilic polymer that can absorb and retain larger amounts of water or biological fluids while maintaining its structure due to chemical or physical cross-linking. They have been used practically for years due to their ability to maintain a moist healing environment, which is beneficial for tissue repair. They are biocompatible and have the property of absorbing wound exudates, which helps keep the wound area clean and enhances wound healing. Although on one side hydrogels are beneficial for keeping the moist environment, this property can also be a high-risk factor in the case of some conventional hydrogels, due to the encouragement of the growth of bacteria and pathogens, which is facilitated by their moist environment, leading to infections. Additionally, due to its bulky character, it can also lead to restricted movement,^35^ these limitations can be overcome by using xerogels. The xerogels provide a drier wound environment compared to hydrogel, as they absorb just a little moisture as required to maintain a moist environment and hence retard the growth and proliferation of pathogens, leading to a reduction in infection. Further, the xerogels are lighter in weight due to their lower water content, making them more comfortable and easier to handle and use as they avoid the movement restriction caused by the hydrogels. The lower water content not only makes it feasible for application but also retards degradation and enhances its stability compared to hydrogels. It should be noted, however, that some advanced hydrogel formulations incorporate antimicrobial or antifouling agents, which effectively counteract the risks associated with microbial growth. Thus, while xerogels offer drier microenvironments and alternative diffusion regimes that may mitigate infection risks, both hydrogels and xerogels have complementary roles, depending on the intended therapeutic context and formulation design.

### 3.3 Fabrication Techniques of Xerogels

Xerogels come in various forms and can be created by using diverse fabrication methods. The most common method is the sol–gel method. In this method, the system transitions from a colloidal liquid, or “sol”, to a solid, or “gel”, phase. This sol–gel method includes three significant steps, starting with hydrolysis and condensation, where precursors like metal alkoxides or organic monomers undergo reactions to form a network of interconnected particles.^12^ This process is followed by gelation, in which the sol transforms into a gel, a semisolid state where the liquid phase is entrapped within a solid network. Then, the gel is aged to strengthen the network and reduce the liquid content.^38^ After the gels are formed, the second step of drying starts, in which the damp gel is dried to remove the liquid phase. The method of drying has a significant effect on the properties of the resulting xerogel. In the case of xerogel, ambient drying is recommended, where the gel is dried under ambient conditions, resulting in the formation of xerogels. This method often results in some shrinkage and densification of the gel network.^39^ The most common form of xerogel is silica xerogel. The formation of silica xerogel through the sol–gel process involves a sequential series of chemical reactions that transform silicon alkoxide precursors into a 3D porous network. Initially, hydrolysis of silicon alkoxide compounds, typically tetraethyl orthosilicate (TEOS), occurs through the reaction Si (OR)_4_ + H_2_O → Si (OR)_3_(OH) + ROS, where water molecules attack the silicon center to produce silanol groups while releasing alcohol as a byproduct. This is followed by condensation reactions, including both silanol condensation [2Si­(OR)_3_(OH) → (RO)_3_Si–O–Si­(OR)_3_ + H_2_O] and alkoxylation, which create Si–O–Si bridges and establish the initial cross-linked siloxane network with concurrent water elimination. During the aging process, unreacted silanol groups continue to react with remaining alkoxide functionalities through the reaction SiOH + SiOR → Si–O–Si + ROH, further strengthening the network connectivity and enhancing the mechanical properties. As the reactions proceed, extensive cross-linking occurs, forming a robust 3D framework where silicon atoms are interconnected through siloxane bridges, resulting in increased rigidity and the development of porosity. The final drying step, conducted under ambient pressure, removes residual solvents and water from the pore structure. Capillary forces cause some network shrinkage while preserving the porous architecture. This controlled transformation from liquid precursors to solid xerogel produces materials with high surface area and tunable porosity, making them valuable for applications in catalysis, adsorption, and advanced materials engineering. The steps of silica xerogel synthesis are shown in Figure 4.^40,41^ Other methods for biomedical applications are also explored to overcome the limitation of sol–gel methods, such as the ionotropic cross-linking method,^42,43^ lyophilization method,^44^ solvent displacement method,^45,46^ facile method (alkali freezing and ambient drying method),^47,48^ and microwave drying method.^49^ Table 2 summarizes the various methods of xerogel preparation and their advantages, applications, and challenges. Figure 5 summarizes the other methods involved in xerogel synthesis. Out of all these methods, the ionotropic cross-linking methods and the lyophilization technique are highly explored.

**Figure 4:**
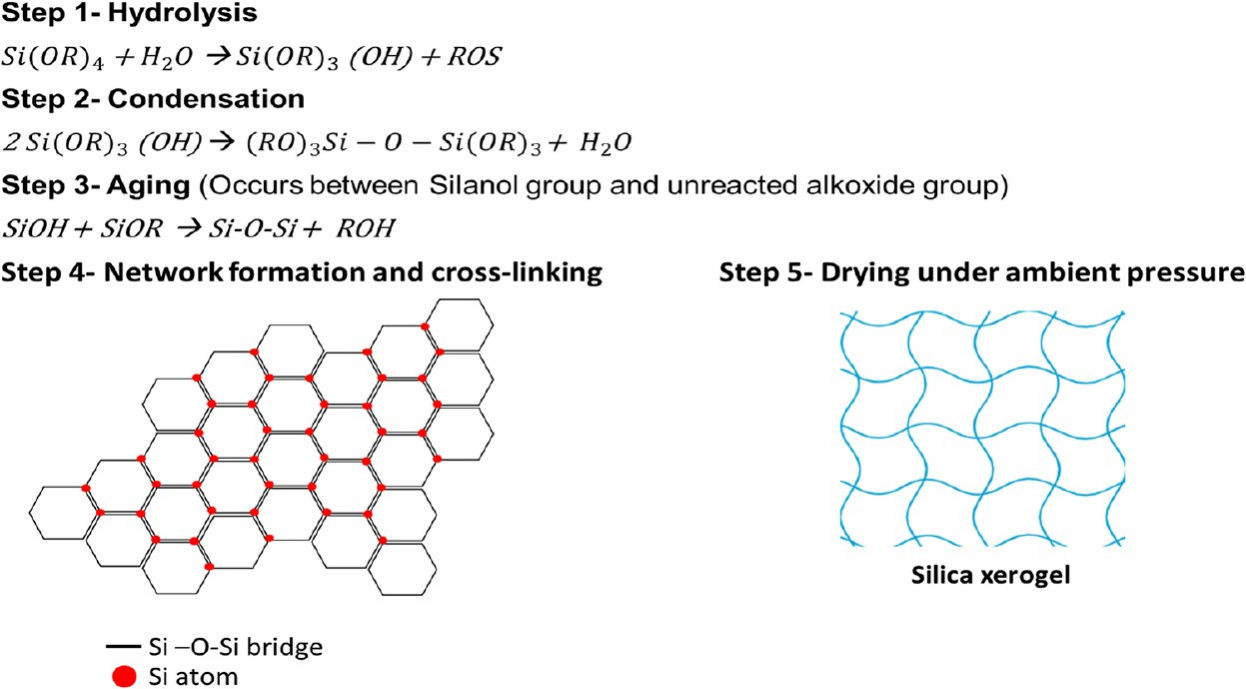
Method of formation of silica xerogel.

**Figure 5:**
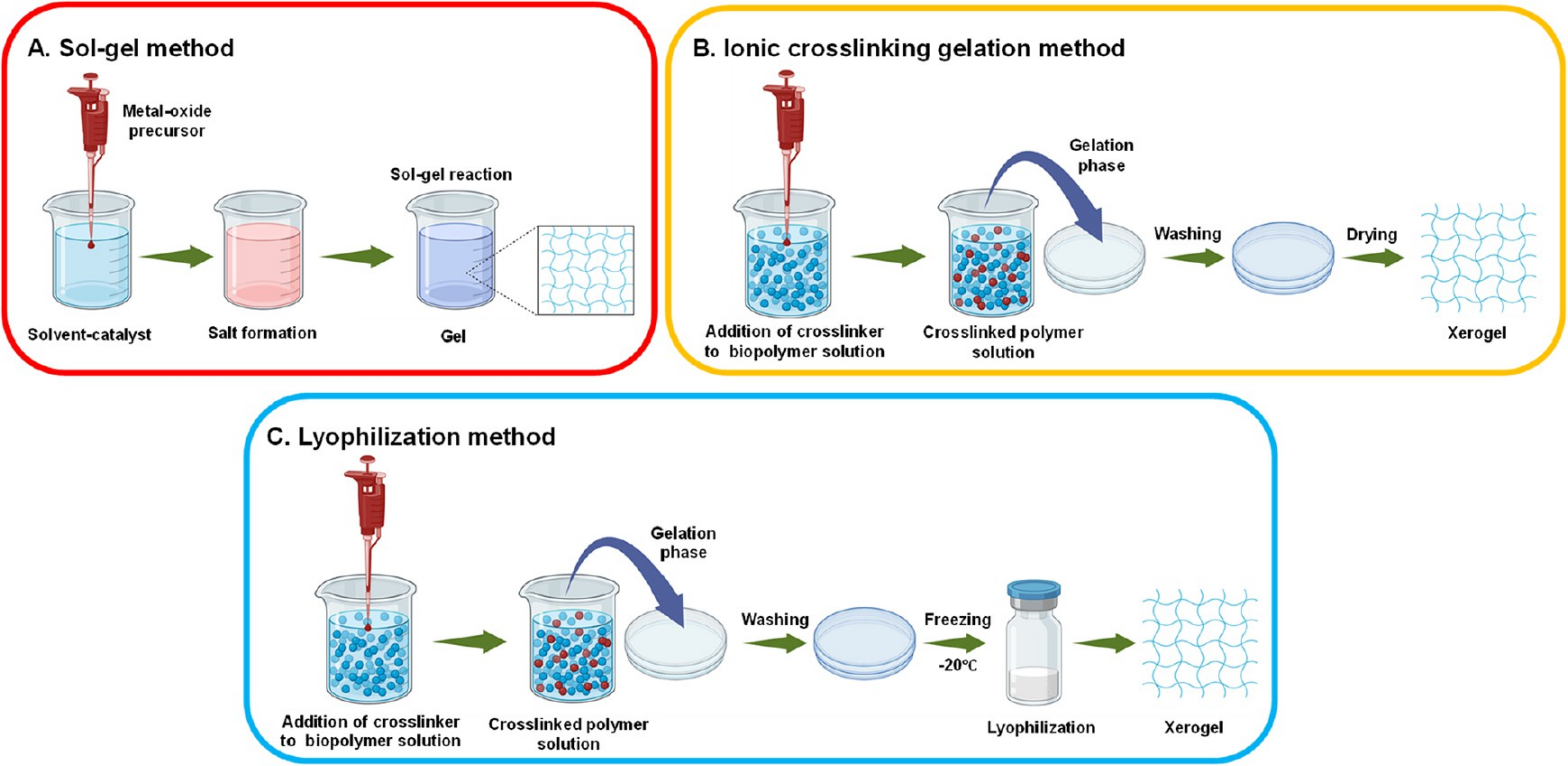
(A) Sol–gel method of preparation of xerogels, (B) ionic cross-linking gelation method, and (C) freeze-drying or lyophilization method.

**Table 2:** Different Methods of Xerogel Preparation, Advantages, Applications, and Challenges

Method	Mechanism	Advantages	Applications	Challenges
Ionotropic Cross-linking Method	Involves the interaction of charged polymer chains with oppositely charged ions to form a three-dimensional network through an ionic bond	Mild and straightforward conditions, suitable for sensitive biological material	Drug delivery systems, tissue engineering	Difficult to control the release kinetics of cross-linking ions
Freeze-Drying (Lyophilization)	Freezes the gel and sublimates ice directly to the gas	Preserves porous structure, minimal shrinkage	Drug delivery, tissue engineering	Time-consuming, energy-intensive
Microwave Drying	It uses microwave radiation to heat and evaporate solvents rapidly	Fast, energy-efficient	Catalysis, adsorption	It may cause uneven drying and require precise control
Alkali Freezing and Ambient Drying	Treats fibers with alkali, freezes, and dries at ambient conditions	Environment-friendly, cost-effective	Biomedical applications, encapsulation	It may require optimization for different materials
Solvent Displacement	Replaces the solvent in the gel with another solvent of lower surface tension	Reduces capillary forces, minimizes shrinkage	Porous materials for catalysis, energy storage	Requires careful selection of solvents

Ionotropic cross-linking is a specific cross-linking method that uses ionic interactions between oppositely charged ions and polyelectrolytes to form gel networks. This is a gentle, reversible process that is commonly used in pharmaceutical and biomedical applications. The polyelectrolytes (polymers with charged groups) interact with multivalent ions of opposite charge. The ions act as cross-linking agents by binding to multiple polymer chains simultaneously. The electrostatic attractions between the charged polymer groups and the cross-linking ions form a 3D network structure without the need for covalent bond formation. Since the cross-links are ionic rather than covalent, they can be disrupted by changes in pH, ionic strength, or the presence of competing ions. Some of the examples of ionotropic cross-linking methods include cross-linking between alginate (containing carboxyl groups) with calcium ions (Ca^2+^). The calcium ions coordinate with the carboxyl groups on different alginate chains.^43,50^ The cross-linking of chitosan with polyanions, such as tripolyphosphate (TPP), etc.^51^ The ionotropic method is particularly valuable for creating xerogels from natural polymers under gentle conditions, making it ideal for applications requiring biocompatibility and the preservation of sensitive encapsulated materials.

In one study, a cellulose xerogel was synthesized by using a straightforward three-stage process that involves selectively dissolving cellulose fibers in an ionic liquid, cleaning without the use of additional solvents, and final dehydration. The methodology begins with controlled dissolution, where cellulose suspension undergoes partial breakdown in an ionic liquid medium, creating a gel-like network while preserving the original fiber architecture. This is followed by a solvent-free washing step that removes excess ionic liquid through an aqueous treatment, thereby maintaining the integrity of the gel structure. The process concludes with ambient drying to eliminate residual moisture and form the final porous xerogel material. This approach offers a simple yet effective route to produce cellulose-based xerogels with minimal environmental impact and processing complexity.^52^

Lyophilization, commonly known as freeze-drying, is a sophisticated dehydration technique that has gained considerable attention in xerogel formulation. This method involves freezing a gel, followed by sublimation of the frozen solvent under vacuum and potentially ambient drying to achieve xerogel characteristics. Lyophilization operates on the principle of sublimation, where frozen solvent (typically water) transitions directly from solid to vapor phase without passing through the liquid state.^53^ Several studies have been carried out to prepare xerogels using this technique. One of the researchers developed porous xerogels from standard and thiolated chitosan by freeze-drying gels containing glycerol, mannitol, and 50% BSA, followed by annealing. The gels (1% TG-chitosan) were stirred to ensure uniformity, then molded and freeze-dried using a monitored cycle. Xerogels from dialyzed TG-chitosan were compared to those from lyophilized powder. Standard chitosan xerogel absorbed more water (1110%) than thiolated (480%), but thiolated xerogels showed better mucoadhesion and higher drug release (94.4% vs 91.5%), indicating potential for buccal protein delivery.^54^ Apart from this, Jiang et al. prepared three-dimensional SnO_2_/rGO xerogels by using a freeze-drying-assisted method. In which graphite oxide was dispersed in ethanediol, combined with SnCl_2_·H_2_O and ethylenediamine, and then heated in an autoclave at 180 °C for 24 h. The resulting gel was washed, freeze-dried, and heat-treated at 400 °C under nitrogen to enhance SnO_2_ crystallinity. The xerogels featured a porous structure with well-dispersed 5 nm SnO_2_ nanoparticles on graphene sheets. They exhibited excellent electrochemical performance, delivering high capacity and stability in both lithium-ion and sodium-ion batteries, making them strong candidates for energy storage applications.^55^

### 3.4 Factors Governing the Structure of Xerogels

Several parameters govern the structure of xerogel, starting from its synthesis conditions, followed by the aging process and drying parameters.^56^

#### 3.4.1 Synthesis Parameter

The synthesis parameter that affects the structure of xerogels includes changes in pH, precursor concentration, and dilution ratio. As per the study carried out by Hasanuzzaman and Tanha^57^ as the pH changes from pH 3 to 4.5, the average pore size of the xerogels was found to be around 31 nm, resulting in mesoporous xerogels with uniform distribution. In contrast, as the pH increases from 6 to 7, the pore sizes increase with an average size of 59 nm, yielding macroporous xerogels. It can be stated that the pH of the solution during the synthesis process has a remarkable effect on the pore size due to its influence on the rate of hydrolysis and condensation. The rate of hydrolysis increases at lower pH values, resulting in the formation of smaller pore size xerogels, whereas the rate of polycondensation surpasses the rate of hydrolysis at higher pH values, resulting in larger pore size formation^57^

The precursor concentration during the synthesis phase considerably affects the structure of the xerogels. As the concentration of the precursor increases, the pore size decreases, forming a uniform surface. This may be attributed to the rise in the gelation rate and the creation of more densely cross-linked networks with increased connectivity between particles or polymer chains. Rosales et al. prepared a hybrid silica xerogel and studied the effect of the organic precursor and its concentration on the porosity and surface chemistry of the xerogels. As per Figure 6(B), it can be observed that as the concentration of triethoxy­(p-tolyl)­silane (MPhTEOS) increases from 1 M to its maximum level, significant structural transformations occur in the xerogel. The initial 1 M sample displayed a stratified morphology characterized by multiple overlapping sheets with relatively smooth surfaces. In contrast, the high-concentration sample develops a glassy, continuous surface interrupted by fracture lines—evidence of tensile forces active during the syneresis phase of gel formation. Specifically, the structural transition involves simultaneous micropore proliferation coupled with mesopore depletion. The higher organic content drives the formation of sub-2 nm pores while eliminating the 2–50 nm pore network, fundamentally altering the surface topography toward increasingly planar morphologies.^17,58^

**Figure 6:**
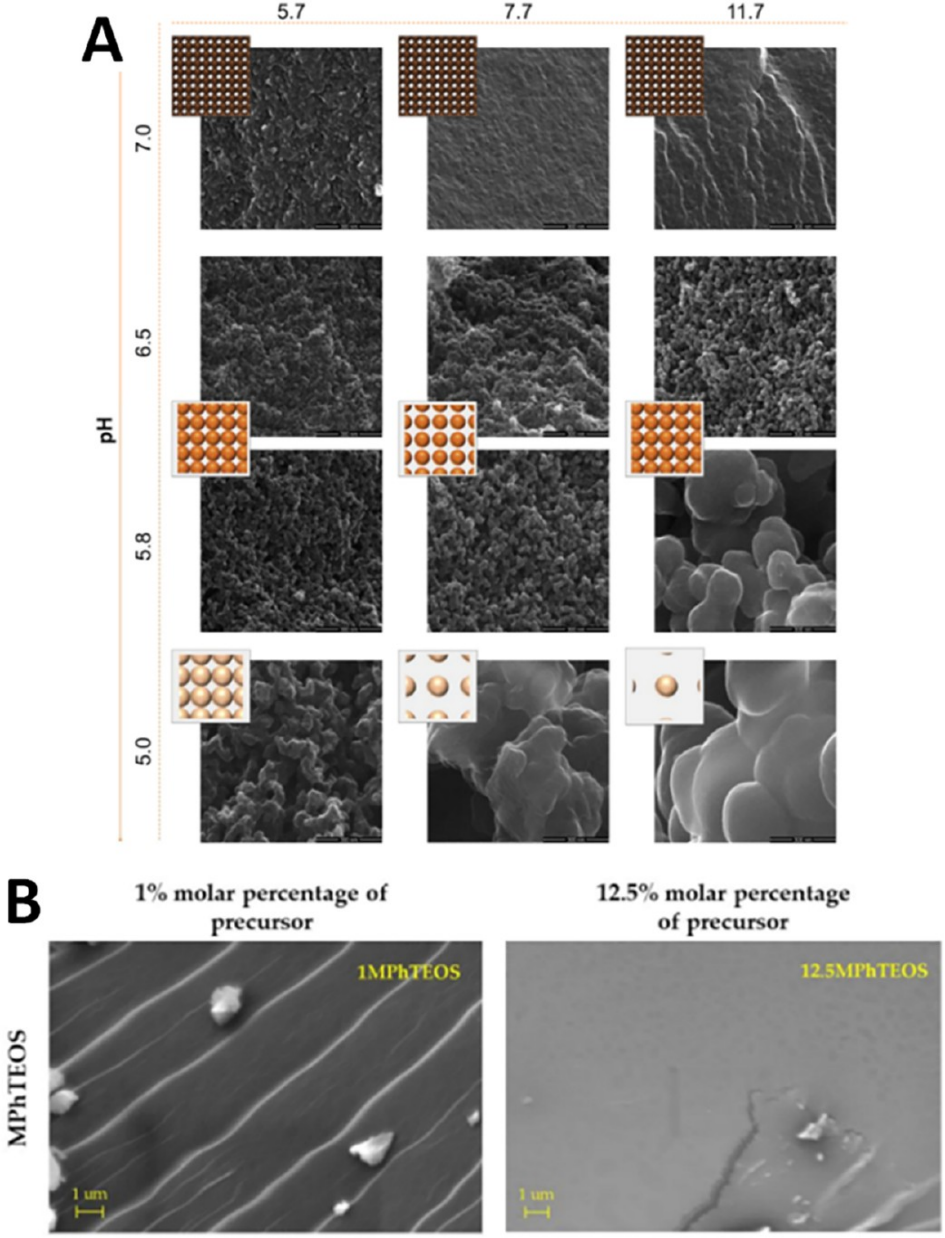
(A) SEM micrograph of carbon xerogel at different pH and Dilution ratios. Adapted with permission from ^59^Copyright © 2015 Elsevier Inc. All rights reserved. (B) SEM micrographs of 1% molar MPhTEOS and 12.5% molar concentration of MPhTEOS. Adapted with permission under a Creative Commons CC BY 4.0 license from.^17^ Copyright 2023, MDPI, Basel, Switzerland.

The dilution ratio (molar ratio of solvent to reactants) also significantly influences xerogel formation and the final properties through several mechanisms. This parameter directly affects the structural integrity and robustness of the Xerogel network. Consequently, this relationship governs the extent of dimensional contraction throughout the drying process as the dilution ratio fundamentally determines how the material responds to stress during solvent removal. A higher dilution ratio yields xerogels with a higher average pore size and lesser mechanical strength due to an increase in shrinkage during drying compared to a lower dilution ratio, yielding densely packed xerogels with better structural and dimensional stability. As per the experiment carried out by Rey-Raap et al., they synthesized an organic xerogel composed of resorcinol and formaldehyde. In the experiment, deionized water was used as a solvent and sodium hydroxide was used as a catalyst. They used three different dilution ratios, ranging from 5.7 to 11.7, with different pH values ranging from 5 to 7. As per the results, it was observed that the pore size increases with an increase in the dilution ratio, and the same can be seen in Figure 6(A).^58,59^

#### 3.4.2 Aging Conditions

The second aspect is the aging conditions; it is the duration for which no further process is carried out or, in other words, the molecules are allowed to sit for further reaction to occur independently of any steps. Aging time and temperature are two aspects of aging conditions that play a crucial role in the structure of the xerogel. In the case of aging time, as it increases, the pore size also increases. At an aging time of 2 days, it was observed that the pore size of xerogel fell in the mesoporous range; i.e., it was found to be around 30 nm. As the time increased from 4 to 6 days, the pore size increased, falling in the range of 80 to 90 nm, i.e., coming under the category of microporous particles. Extended aging promotes Ostwald ripening, where smaller particles dissolve and redeposit onto larger particles, affecting the pore size distribution and connectivity. Longer aging times allow for more extensive condensation reactions and cross-linking, resulting in stronger gel networks that better resist shrinkage during drying. Apart from the aging time, the temperature at which the gel is aged also affects the structure of xerogels. Higher aging temperatures accelerate condensation and cross-linking reactions, potentially compressing the aging time requirements. Optimized aging protocols (combination of temperature and time) can reduce drying-induced shrinkage by up to 40%.^57,60^

#### 3.4.3 Drying Condition

Xerogels are formed when the liquid component of a gel is removed through evaporation. The drying conditions significantly influence the final xerogel structure through several mechanisms. During drying, surface tension at the liquid–gas interfaces creates capillary pressures that can cause significant structural collapse. Faster drying rates typically create higher capillary pressures, leading to more substantial shrinkage and densification. Slow, controlled drying often produces more uniform structures, with fewer cracks and defects. Rapid drying can create significant stress gradients within the gel, leading to cracking, warping, and nonuniform properties. The considerable difference between xerogels and aerogels is their method of drying. In the case of aerogels, supercritical drying is done, whereas in the case of xerogels, an ambient drying protocol is used. One supercritical fluid, such as carbon dioxide, is used in supercritical drying. The capillary pressure is reduced in this case due to the nonexistence of a liquid–gas interface, leading to minimal shrinkage, and hence, it offers a higher surface area. Whereas in the case of ambient condition drying, the evaporation occurs at ambient pressure, leading to increased capillary pressure, and hence, there is more shrinkage and a greater chance of cracks, which can be controlled by controlling the conditions.^36,61^ The highlights of each parameter are listed in Table 3.

**Table 3:** Parameters Governing the Xerogel Structure

Parameter	Effect on structure	Mechanism	Key findings	Refs
1. Synthesis Parameters				
pH	Controls pore size distribution	Lower pH: Faster hydrolysis rate	pH 3–4.5: ∼31 nm pores (mesoporous)	^57^
		Higher pH: Faster polycondensation rate	pH 6+: ∼59 nm pores (macroporous)	
			pH critically affects the hydrolysis vs condensation balance	
Precursor Concentration	Affects pore size and surface uniformity	Higher concentration → increased gelation rate	Higher concentration: Smaller pores, uniform surface	^58^
		Creates densely cross-linked networks	Structural transition: Micropore proliferation with mesopore depletion	
		More connectivity between particles	Glassy, continuous surface at high concentrations	
Dilution Ratio	Influences structural integrity and mechanical properties	Higher ratio → more shrinkage during drying	Higher dilution: Larger pores, lower mechanical strength	^58^
		Lower ratio → densely packed structure	Lower dilution: Better structural and dimensional stability	
		Governs dimensional contraction during drying	Direct relationship between dilution and pore size	
2. Aging Parameters				
Aging Time	Controls pore size growth	Extended aging promotes Ostwald ripening	2 days: ∼30 nm (mesoporous)	^60^
		Allows extensive condensation reactions	4–6 days: 80–90 nm (microporous)	
		Increases cross-linking	More extended aging creates stronger gel networks that resist shrinkage	
Aging Temperature	Accelerates structural development	Higher temperature → faster condensation	Optimized protocols: Reduce drying shrinkage by up to 40%	^57,60^
		Accelerated cross-linking reactions	Temperature can substitute for an extended aging time	
		Can compress aging time requirements	Critical for controlling the final structure	
3. Drying Parameters				
Drying Rate	Controls structural integrity and defects	Fast drying → higher capillary pressure	Fast drying: More shrinkage, densification, stress gradients	^61,62^
		Slow drying → more uniform structure	Slow drying: Fewer cracks, better uniformity, fewer defects	
		Surface tension creates capillary pressures	The rate directly affects the final quality	
Drying Method	Determines final porosity and surface area	Ambient: Liquid–gas interface, higher capillary pressure	Xerogels (ambient): More shrinkage, lower surface area	^36,61^
		Supercritical: No interface, minimal pressure	Aerogels (supercritical): Minimal shrinkage, higher surface area	
			Method choice defines material classification	

## 4 Xerogel for Wound Healing Application

### 4.1 Mechanism of Action of Xerogels in Wound Healing

Xerogels represent a revolutionary advancement in wound care technology, demonstrating extraordinary wound healing capabilities through their sophisticated multimodal absorption mechanisms that leverage their huge surface area-to-volume ratios and hierarchically organized enhanced porosity to effectively manage wound exudates while maintaining the delicate optimal moisture balance essential for proper wound healing environments.^63^ The intricate interconnected pore structure of xerogels, characterized by micro-, meso-, and macroporous networks, facilitates rapid and efficient diffusion of complex wound exudate molecules containing proteins, inflammatory mediators, and cellular debris into the xerogel matrix through capillary action and molecular sieving effects, thereby preventing harmful fluid accumulation that could otherwise lead to tissue maceration, delayed healing, or opportunistic bacterial colonization and biofilm formation,^64^ ultimately helping maintain optimal wound hydration levels while simultaneously preventing infection and creating a physiologically favorable healing microenvironment that supports natural regenerative processes.

Advanced xerogels incorporated with carefully selected antimicrobial agents demonstrate remarkably potent and broad-spectrum bactericidal mechanisms through their expansive surface area providing countless active sites for direct antimicrobial interaction and bacterial membrane disruption, while their precisely engineered porous structure allows for controlled and sustained release of antimicrobial compounds at therapeutically effective concentrations over extended periods, with complex electrostatic interactions between negatively charged bacterial cell walls and strategically modified positively charged xerogel surfaces contributing significantly to bacterial capture, immobilization, and subsequent elimination through multiple cytotoxic pathways including membrane permeabilization and oxidative stress induction.^65–67^

Xerogels formulated using biopolymers such as chitosan and gelatin, actively promote rapid and effective hemostasis through their ability to enhance platelet activation cascades and accelerate thrombin production pathways, with their three-dimensional porous structure providing an optimal physical scaffold that facilitates platelet aggregation, adhesion, and activation while concentrating clotting factors at the wound site.^68^ Xerogel-based bioadhesives significantly enhance hemostatic efficacy by overcoming the limitations posed by rapid, pressurized blood flow during hemorrhage, which typically compromises conventional agents and sealants. The macroporous, tough xerogel rapidly absorbs interfacial fluids such as whole blood, accelerating clot formation. Its infusion with functional liquids promotes strong interfacial bonding, effective sealing, and antibacterial action. This synergistic design enables robust adhesion to biological tissues and engineered surfaces without requiring compression and offers instant removability and long-term storage stability. Compared to nonstructured and commercial alternatives, these xerogel bioadhesives demonstrate superior hemostatic performance and biocompatibility in animal models, paving the way for advanced wound care solutions.^69,70^

The comprehensive mechanism of tissue regeneration involves xerogels functioning as highly sophisticated biocompatible scaffolds that actively promote and guide essential cellular activities throughout all phases of wound healing, with their carefully engineered porous architecture providing a biomimetic three-dimensional framework that closely resembles native extracellular matrix structure and actively supports directed cell migration, controlled proliferation, and guided differentiation of multiple cell types including fibroblasts, keratinocytes, endothelial cells, and immune cells, while the precisely tuned surface chemistry of xerogels, particularly those intelligently derived from bioactive natural polymers such as chitosan with its inherent antimicrobial properties,^72^ alginate with its excellent gelation characteristics,^73^ or modified cellulose with its superior mechanical properties,^74^ facilitates optimal cellular adhesion, spreading, and growth through specific integrin-mediated interactions and growth factor binding. The dynamic xerogel matrix maintains an ideal moist wound environment with controlled water activity that actively encourages rapid keratinocyte migration and proliferation for effective re-epithelialization, while simultaneously promoting fibroblast proliferation and collagen synthesis essential for robust wound closure and functional tissue repair, with the strategically designed interconnected pore network allowing for efficient nutrient diffusion, oxygen transport, and metabolic waste removal, thereby supporting optimal cellular metabolism and energy production during the intensive healing process while preventing the accumulation of toxic metabolites that could impair healing.

Xerogels function as controlled drug delivery systems through their precisely engineered porous structure and carefully modified surface chemistry, with diverse therapeutic agents, including broad-spectrum antibiotics,^66^ specific growth factors, anti-inflammatory compounds, analgesics, and wound healing accelerators being strategically incorporated into the xerogel matrix through various loading techniques including physical entrapment, chemical conjugation, and electrostatic binding, where multiple simultaneous processes including molecular diffusion govern the complex release mechanism through the tortuous pore network, controlled degradation of the xerogel matrix through enzymatic or hydrolytic pathways, and competitive desorption from active surface sites.^75,76^ This sophisticated multimodal controlled release mechanism ensures sustained and therapeutically effective concentrations of active compounds are maintained at the wound site over extended periods, significantly reducing the frequency of painful dressing changes and dramatically improving patient compliance and comfort while maintaining optimal healing conditions and preventing the development of drug resistance through consistent therapeutic dosing. The mechanism of action of xerogels is depicted in Figures 7 and 8.

**Figure 7:**
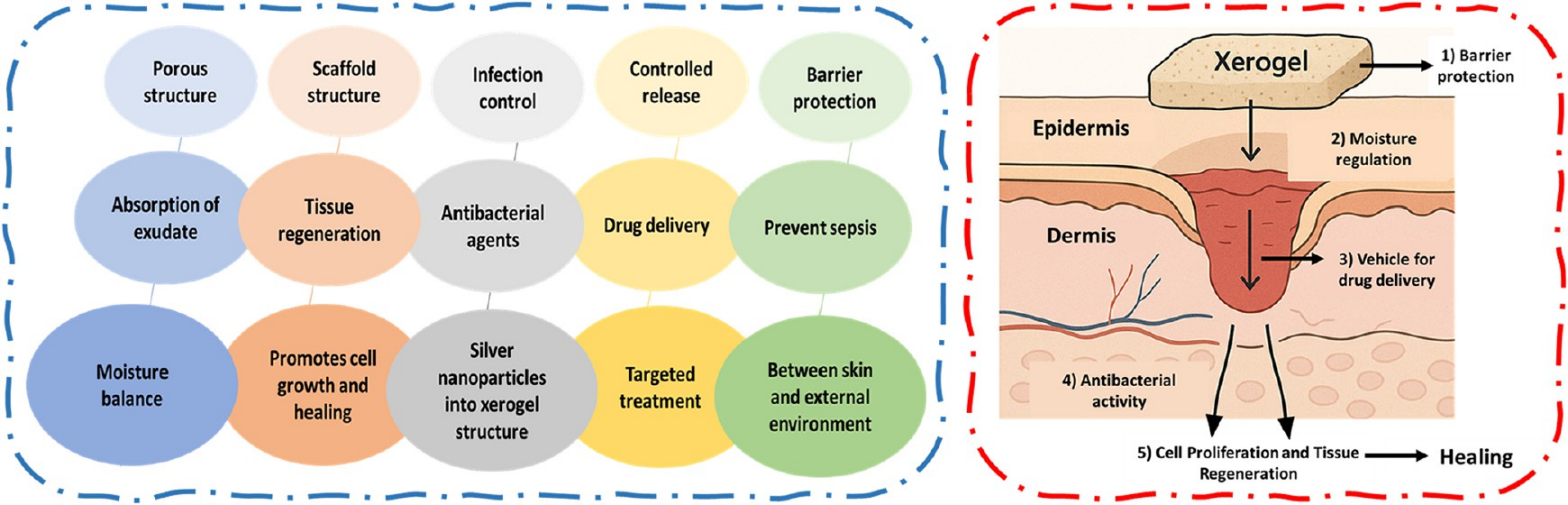
- Schematic representation of various mechanisms of xerogels in wound healing.

**Figure 8:**
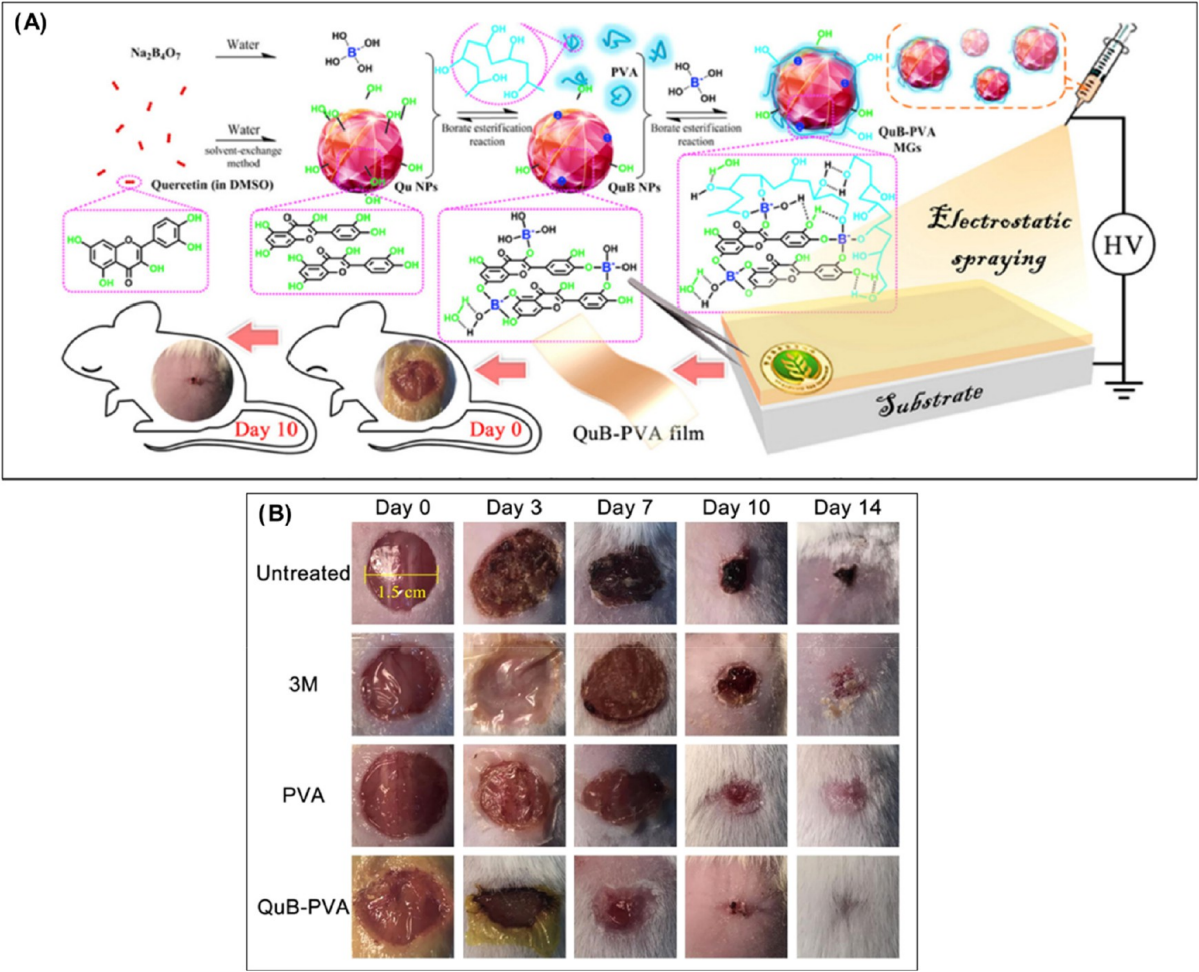
(A) Working mechanism of Quercetin Borate PVA xerogel film in wound healing. (B) The wound healing effect of the Quercetin Borate PVA xerogel film is compared to that of different wound healing dressings. Adapted under the permission of ref 65 under © 2021 Elsevier B.V. All rights reserved.

### 4.2 In Management of Diabetic Wound

Diabetic foot ulcers are open lesions or sores that typically occur on the bottom of the foot in individuals with diabetes. They are a common and severe complication of diabetes, often resulting from a combination of neuropathy (nerve damage), poor circulation, and infection. It can lead to severe infections, gangrene, and even amputation if not correctly managed.^77^ Different topical dressings are available for managing diabetic foot ulcers including alginates, foams, and hydrogels. However, before starting treatment, it is crucial to understand the local wound’s microenvironment, as it influences the wound-healing process. The wound dressings should possess essential characteristics such as controlling local inflammatory responses, glucose levels, high protease activity, and regulating reactive oxygen species (ROS) formation at the wounds.^78^ Rajalekshmy et al. formulated APEG-g-poly (PEGMA) xerogels loaded with simvastatin (SIM) and strontium (Sr). These xerogels were tested on L929 fibroblast and HaCaT keratinocyte cells under high glucose conditions. ADPM2S loaded with Sr and SIM demonstrated sustained release of Sr ions (55%) and simvastatin (SIM, 73%) over 24 h, achieving therapeutic levels of 3 mM and 60 μM, respectively. SIM-loaded ADPM2S enhanced anti-inflammatory activity to 47%, compared to 32% with ADPM2S alone, and significantly increased collagen production in fibroblasts. Wound closure reached 67% in L929 cells and 45% in HaCaT cells within 8 h. SIM also promoted macrophage polarization toward an anti-inflammatory phenotype, up-regulated genes related to collagen synthesis and cell migration, and modulated cytokine expression by down-regulating TNF-α and IL-6 while up-regulating IL-10, supporting its role in orchestrating wound healing.^79^ It has been observed that enzyme-incorporated dressings offer an innovative approach to wound care, aiming to enhance the healing microenvironment through continuous enzyme delivery. Key development factors include localized delivery, extended activity duration, structural integrity, and optimal pH for enzyme function. However, the high levels of ROS, persistent inflammation, and protease activity in chronic wounds can negatively impact enzymatic performance, and this issue has yet to be fully resolved in advanced wound care products.^80^ Hence, to overcome this limitation, Rajalekshmy et al. prepared a xerogel wound dressing material loaded with glucose oxidase and peroxidase enzymes (GO-POD). The prepared xerogel (ADPM2S) exhibited favorable physicochemical properties, with a swelling rate of 1500%, a tensile strength of 400 kPa, and a water vapor transmission rate of 1490 ± 76 g/m^2^/24 h. As per the scratch assay performed on fibroblast cell lines, the cells treated with the GO-POD-loaded xerogel showed a significant enhancement in wound closure (57%) compared to untreated cells (20%) within 8 h in a high glucose medium, as can be observed in Figure 9. Increased collagen deposition and enhanced migration of fibroblast cells were also observed. Hence, it can be concluded that the alginate xerogels loaded with GO-POD have a promising approach for diabetic wound management.^81^ They have also studied the wound healing effect of alginate-methacrylate xerogel for delivery of insulin using scratch assay method on keratinocyte cell line and have found that the xerogel formulation not only increased the physical stability but has also shown that approximately 70% of insulin was released from xerogel throughout 48 h which had a positive impact on wound healing and suggested its future application in wound care management.^44^ The authors further studied the topical delivery of insulin loaded in alginate diamine PEG-*g*-poly­(PEGMA) (ADPM2S2) xerogels using diabetic rats. The xerogel was able to release insulin for a period of 48 h and maintain its biological activity, along with its structural stability. The in vivo study demonstrated that approximately 95% of wound closure was achieved within 14 days of treatment, compared with 82% in the control group. Whereas, as per different types of in vitro studies performed using various cell lines, it was concluded that insulin-loaded xerogels demonstrated remarkable enhancement in cell migration, proliferation, and collagen deposition when compared to control cells under increased-glucose conditions and promoted wound healing.^82^ Hence, the xerogels can be quickly loaded with both drugs and enzymes, thereby improving diabetic wound healing. It is further observed that although excellent results have been achieved in diabetic wound healing, the number of studies is significantly limited. Hence, this area can be further studied by researchers to enhance diabetic wound care management.

**Figure 9:**
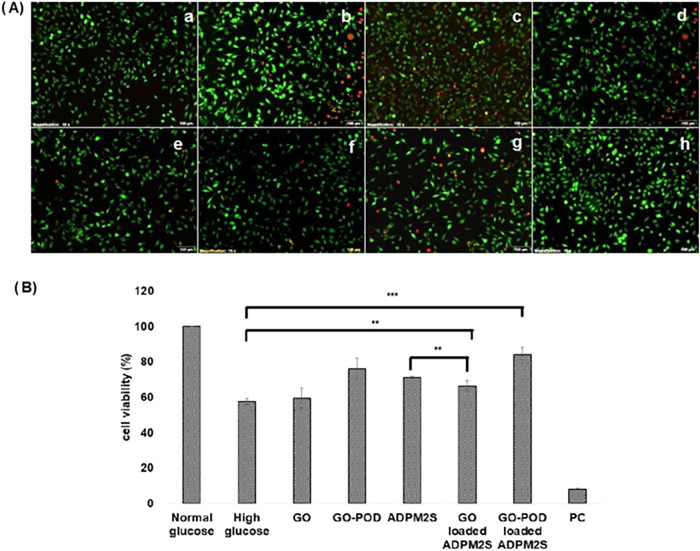
(A) Cell line study results performed on L929 fibroblast cells (a) indicating control, (b) media with high glucose level, (c) hydrogen peroxide, (d) glucose oxidase (GO), (e) glucose oxidase peroxidase (GO-POD), (f) ADPM2S, (g) glucose oxidase loaded ADPM2S, and (h) glucose oxidase peroxidase loaded ADPM2S and (B) MTT assay performed in fibroblast cells.^81^ Adapted with permission from © 2022 Acta Materialia Inc. Published by Elsevier B.V. All rights reserved.

### 4.3 Bacterial Wound Management

Dealing with bacterial infections in wounds is challenging because bacteria can develop biofilms on wound surfaces, making them resistant to antibiotics. The body’s immune defense^83^ and the persistent bacterial presence can lead to chronic inflammation, delaying healing and increasing the risk of complications. Wounds have a complex microenvironment with varying pH levels, oxygen availability, and nutrient availability, which affects bacterial growth and treatment efficacy. Xerogels loaded with antibacterial agents can effectively address the above issues, as, due to their superhydrophobic and excellent moisture absorption properties, they can keep the wound area dry, preventing the growth of pathogens.^84^ For example, Huang et al. assesed the antibacterial activity of the new AgNPs/N-CD@ZnO PTLA photoresponsive xerogel (P2), which was tested against S. aureus and *E. coli* (10^6^ CFU/mL) in both the dark and under 808 nm near-infrared irradiation conditions for 15 min at 1 W cm^–2^. For comparison, a control xerogel without a photoactive and silver component was prepared (P1). In the dark, P2 exerted a moderate inhibitory effect (∼37% reduction) due to the limited release of Ag^+^ ions. Under NIR irradiation, however, strong generation of ROS from N-CD@ZnO was induced, which synergistically interacted with Ag^+^ ions and killed bacteria by 99.9% within 15 min. SEM images revealed the loss of membrane integrity and the disintegration of the bacteria. The photoresponsive xerogel demonstrated rapid bacterial capture, effective light-activated bacterial killing, and outstanding wound-healing capabilities, proving to be a potent next-generation antibacterial wound dressing. It was studied for its antibacterial and rapid bacterial killing effects using photodynamic therapy.^85^ The wounds are always accompanied by inflammation, so any dressing that can not only deliver antibacterial agents but also load a multidrug with different pharmacological activities will enhance wound healing. Drozdov et al. developed a magnetite xerogel biocomposite containing four drugs: chlorhexidine digluconate, lidocaine, prednisolone, and chymotrypsin. This combination enhanced wound healing rates by about 1.5 times, achieving complete healing in 14 days compared to 21 days for the control group.^86^ Huang et al. synthesized xerogels using click chemistry with lipoic acid to capture bacteria and promote tissue repair. These xerogels, featuring good ductility, self-healing capabilities, and biocompatibility, effectively captured over 60% of *Staphylococcus aureus* with a bacterial count of 10^6^ within 30 min, through strong electrostatic adsorption, and could be used as removable skin patches for treating bacteria-infected wounds in emergencies.^87^ It has been observed that copper plays a significant role in wound healing due to its broad-spectrum antibacterial effects, helping prevent and control wound infections.^88^ It stimulates the development of new blood vessels, which are vital for delivering oxygen and nutrients to the healing tissue. Moreover, it boosts fibroblast growth, which is critical for generating collagen and other extracellular matrix components.^89^ Building on this concept, Xiaohu et al. developed mesoporous copper-doped silica xerogels (m-SXCu) with different Cu contents (1–5 wt %) using a sol–gel method and subsequently tested against *E. coli* and S. aureus. The xerogel specimens were then exposed to a humid environment (>90% relative humidity) at 37 ± 1 °C for 1 and 24 h, respectively. It was found that the pure silica xerogels (m-SX) have negligible antibacterial activity, while the corresponding Cu-doped ones showed evident and dose-dependent bactericidal effects. Specifically, m-SXCu_5_ achieved 99% bacterial reduction within 1 h, while all Cu-containing xerogels reached nearly complete inhibition within 24 h. The improved performance is associated with sustained Cu^2+^ ion release from the mesoporous matrix and electrostatic interactions that damage the bacterial membranes. Thus, the xerogel acted as an effective antibacterial scaffold by leveraging high surface area and controlled Cu ion release for potential applications in wound healing.^90^ Research has shown that not only synthetic drugs but also metal ions and herbal drugs can be easily incorporated into the Xerogel matrix. Karami et al. prepared several composite xerogel formulations of chitosan-silica and tested them in different experimental conditions to evaluate their antibacterial and wound-healing performances. Xerogels were challenged with both *Staphylococcus aureus* and *Escherichia coli* using a standardized bacterial inoculum. Incubation under controlled humidity and temperature simulated specific wound conditions. Several different xerogel formulations, with or without doping or bioactive agents, were compared to investigate the influence of the formulation on the antibacterial efficacy and cell compatibility. The obtained results showed that the xerogels exhibited high antibacterial activity, while samples doped with bioactive metal ions or nanosilica ensured a 95–99% reduction in bacterial growth within 24 h. Moreover, such xerogels improve the in vitro proliferation of fibroblasts and cell-induced wound closure, confirming their double role of antibacterial and biointeractive scaffolds. In summary, the present study pointed out the crucial role of the xerogel matrix, which can provide a high-surface-area porous framework suitable for promoting sustained ion release, moisture retention, and cell adhesion, representing an ideal candidate for treatments involving wounds.^72^ Even quercetin borate nanoparticles loaded in the xerogels increased the antimicrobial property, enhanced the antioxidant and self-healing, and promoted wound healing.^65^ It has been observed that xerogels can be easily converted into powder form, enhancing their handling and stability. A researcher prepared a chlorhexidine-loaded hydrogel through free radical polymerization of sulfobetaine and keratin and later converted it into xerogel by lyophilizing the hydrogel and grinding it into xerogel powders for further studies. These biodegradable xerogel powders are more convenient for sterilization, formulation, and storage. When applied as a powder, the xerogel absorbs wound exudates and transforms into a hydrogel in situ, enhancing its self-healing properties. In vivo studies of septic wounds showed that the xerogel powder dressing significantly increased collagen deposition and reduced inflammation, thereby accelerating wound closure and skin regeneration. These promising materials hold great potential for wound-healing applications.^66^ Deon et al. developed a composite xerogel by immobilizing chitosan-stabilized gold nanoparticles onto a silicon dioxide/titanium dioxide magnetic xerogel. This composite leverages the antimicrobial properties of the nanoparticles, titania’s reactivity, silica’s porosity, and magnetite’s magnetic response. It proved effective against *E. coli*, inhibiting bacterial growth even with low gold content, and maintained its antibacterial properties after magnetic recovery.^91^

### 4.4 As a Hemostatic Agent

Traumatic hemorrhage refers to severe bleeding resulting from a physical injury or trauma. Accidents, falls, sports injuries, and violent events often cause this type of bleeding. Such a type of bleeding can be fatal if not controlled promptly, as severe blood loss can result in shock, organ failure, and, ultimately, death. Effective management involves immediate first-aid measures, such as applying pressure to the wound and advanced medical interventions to stabilize the patient and control the bleeding. Conventional topical hemostatic agents, such as gauze, are insufficient to control severe blood loss. Hence, there is a need for some topical hemostatic materials that can overcome the limitations of conventional systems, such as inflammation, toxicity, etc.^92^ Several hemostatic materials are available in the market, such as Hemcon (chitosan-based), TraumaDEX (potato-starch-based microparticles), and QuickClot (zeolite-based); these are all effective in reducing bleeding time. While these agents possess antimicrobial properties, they also have significant drawbacks, such as poor biodegradability, and they are difficult to remove, which makes them unsuitable for complex wounds. Hence, there is a requirement for the development of an effective and safe dressing to overcome all the above limitations.^93^ To make an ideal hemostatic dressing material, it should have some properties such as stopping bleeding quickly, being easy to apply, being stable, and being biodegradable. It should also possess good absorption properties and show antimicrobial effects to avoid infection. Nowadays, several research studies have been carried out using natural and synthetic polymers such as chitosan, cellulose, hyaluronic acid, alginate, collagen, fibrin, polyurethane, poly­(vinyl alcohol), etc., to make a standard hemostatic material having qualities mentioned above.^94^ Chitosan’s cationic nature and hydrogel-forming properties effectively control bleeding. It helps concentrate erythrocytes and platelets at injury sites, which promotes clot formation. Commercial preparations, such as Celox foam powder and HemCon bandages, utilize these properties for hemorrhage control. However, the effectiveness can vary due to issues with clot stabilization, mechanical strength, porosity, and adherence to wound surfaces. To overcome these limitations of chitosan.^95^ Patil et al. developed a highly porous xerogel as a multimodal topical hemostat by cross-linking chitosan and gelatin with sodium tripolyphosphate. This xerogel, containing synthesized silica nanoparticles and calcium, improved blood clotting 16 times more effectively than Celox and Gauze, as observed in Figure 10. In vivo studies showed that the xerogel composite achieved rapid hemostasis in lethal femoral artery injuries in rats within 2.5 min—faster than commercial Celox (3.3 min) and Gauze (4.6 min), and could be easily removed post-treatment. Additionally, the γ-irradiated xerogel remained stable for up to 1.5 years, indicating excellent shelf life and usability.^42^ Dai et al. formulated macroporous xerogel beads coated with chitosan-containing mesoporous silica (CSSX) with excellent biocompatibility. The in vivo efficacy of the CSSX beads were studied on lethal extremity arterial bleeding on 24 rabbits using standard gauze compression as the control group. The CSSX group showed a 100% survival rate, with an average time to stop bleeding of 95.5 ± 10.1 s, whereas the standard gauze was unable to stop bleeding, and no animal survived at the end. The beads significantly accelerated the coagulation cascade, particularly with 2% chitosan and 5% PEG, achieving effective hemostasis without causing exothermic reactions or tissue thermal injury, and exhibited no cytotoxicity after 7 days.^96^ On the other hand, Qian et al. developed a Sodium polyacrylate (SPA) cochitosan xerogel that can absorb 180 times its weight in water within approximately 3.5 min. The xerogel sponge exhibited exceptional hemostatic properties, outperforming zeolite granules, chitosan granules, and kaolin gauze when tested on a lethal extremity arterial bleeding model in rabbits. It provided effective external pressure and adhered to wet wound tissue, proving to be a rapid and effective first-aid solution for controlling severe hemorrhage in vitro and in vivo.^97^ Another natural material used as a hemostatic agent is silk fibroin. Silk fibroin is gaining attention as a hemostatic agent due to its outstanding biocompatibility, biodegradability, and mechanical properties. It promotes blood clotting by enhancing cell adhesion and reducing bleeding time.^98^ However, the pure silk fibroin hydrogel has a limitation of low water absorption capacity due to the increased cross-linking of the hydrogels. When this cross-linking is reduced, it reduces the gels’ mechanical strength and integrity.^39^ Hence, to overcome this issue and proper utilization of silk fibroin, Cheng et al. formulated highly absorbent silk fibroin protein xerogel by controlling the cross-linking by the addition of peroxidase as a catalyst in hydrogel formation, which prevented the crystal formation and physical entanglement of fibroin leading to the formation of amorphous fibroin hydrogels, which had excellent swelling and water absorption properties. They studied the hemostatic activity of the formed xerogel using a rabbit ear hemostasis experiment. According to the results, it was observed that the hemostatic time taken by Silk fibroin xerogel was 68 s, and the bleeding volume was 0.33g, which was comparatively better than the medical hemostatic gauze, which had a hemostatic time of approximately 197 s and a bleeding volume of 1.12 g. Hence, this xerogel showed great promise for rapidly stopping bleeding and absorbing other body fluids.^63^ There are further requirements of some bioadhesive sealants for the management of noncompressible hemorrhage, which refers to severe bleeding that cannot be controlled by direct pressure, tourniquets, or other compression methods that typically occur in areas of the body where it is difficult to apply pressure, such as the torso, where organs and major blood vessels are located. Bioadhesive sealants physically block bleeding sites but struggle with pressurized blood due to their non- and nanoporous structures. Absorbing and withstanding pressurized blood flows are essential for effective hemostatic treatment in noncompressible hemorrhage.^99^ Bao et al. addressed the limitations of bioadhesive sealants by developing liquid-infused microstructured bioadhesives (LIMBs) using macroporous hemostatic xerogel infused with functional liquids. These bioadhesives absorb fluids and promote clotting, enhancing bonding, sealing, and antibacterial properties. LIMBs form strong adhesions on various tissues and surfaces without compression and have shown superior hemostatic efficacy and biocompatibility in vivo compared to nonstructured alternatives and marketed products.^69^

**Figure 10:**
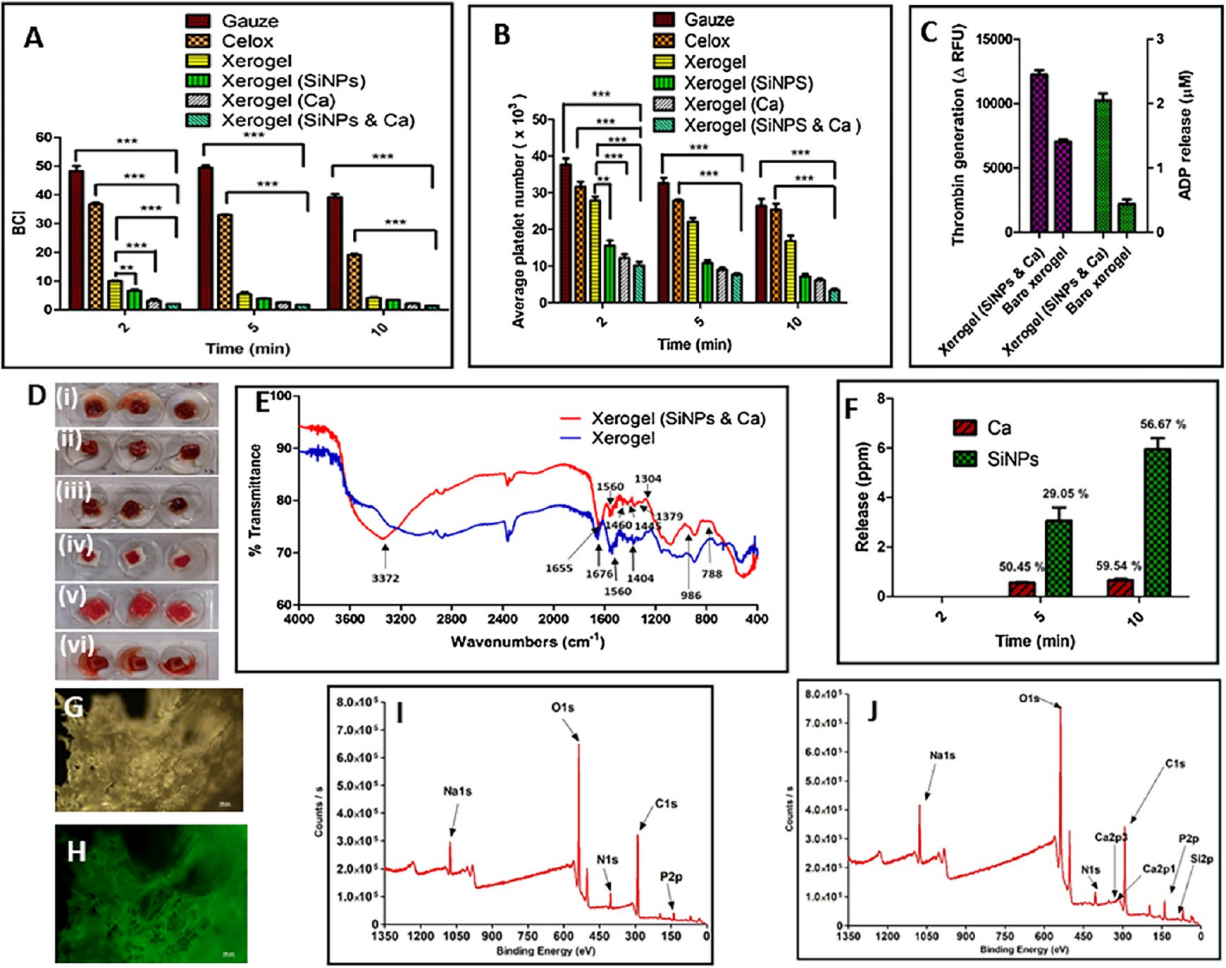
Comparison of hemostatic efficiency of silica nanoparticle (SiNPs) and calcium (Ca) doped Xerogel composite with marketed dressings such as Gauze and Celox. (A) study of blood clotting index (BCI) and (B) aggregation of platelets during hemostasis, (C) thrombin production and platelet activation induced by xerogel composite and bare xerogel, (D) images of blood coagulation with (i) xerogel, (ii) xerogel loaded with silica nanoparticle, (iii) xerogel loaded with calcium, (iv) xerogel loaded with silica nanoparticles and calcium, (v) Celox, and (vi) Gauze. (E) FTIR analysis of xerogel (blue) and xerogel loaded with silica nanoparticles and calcium (red). (F) Si and Ca are released from xerogel by atomic absorption spectroscopy. Distribution of SiNPs in xerogel (G) bright field (H) fluorescence image with FITC-tagged SiNPs 400xXPS analysis of (I) bare xerogel and (J) xerogel loaded with silica nanoparticle and calcium. Adapted with permission from^42^ under © 2020 Elsevier B.V. All rights reserved.

### 4.5 Skin Scaffold and Skin Surface Healing

Skin scaffolds are frameworks crafted to aid in the growth and repair of the skin tissue. They foster an environment that supports cell attachment, development, and differentiation, crucial for regenerating damaged or lost skin.^100^ These scaffolds can be constructed from various materials including natural polymers, synthetic polymers, or a combination of both. They can be designed to replicate the natural extracellular matrix of the skin, playing a crucial role in tissue engineering and regenerative medicine.^101^ Due to their unique properties, xerogels play a significant role as skin scaffolds in tissue engineering. They provide a porous structure that supports cell attachment, proliferation, and differentiation, which are essential for skin tissue regeneration. They can mimic the natural extracellular matrix, promote wound healing, and stimulate angiogenesis (the formation of new blood vessels). Additionally, they can be combined with other materials, such as chitosan, to improve their mechanical strength, porosity, and antibacterial properties.^102^ Elshishiny et al. designed innovative three-layered, asymmetric porous scaffolds that replicate natural skin layers. The upper layer of the scaffold comprises electrospun chitosan-poly­(vinyl alcohol), and the lower layer is composed of xerogels loaded with skin extracellular matrix components. Both layers are joined together by fibrin glue. The scaffold demonstrated excellent swelling capacity for absorbing wound exudates, and it also maintained a stable, degradable weight, making it ideal for burn wounds. The electrospun nanofibrous layer exhibits strong antibacterial properties, effectively inhibiting both Gram-positive (*Staphylococcus aureus*) and Gram-negative (*Escherichia coli*) bacterial strains. Additionally, the scaffolds were found to be biocompatible based on cytotoxicity studies conducted on mouse embryonic fibroblast cells. The in vitro studies further concluded that these scaffolds have an excellent effect on cell proliferation, leading to complete closure of the wound. The study highlights the exceptional biological properties of this novel asymmetrical composite, suggesting it is a promising candidate for clinical applications in replacing burned or damaged skin layers.^71^ Beyond chitosan, collagen is crucial in skin scaffolds due to its unique attributes. It serves as the skin’s structural support, maintaining firmness, plumpness, and moisture retention.^103^

A collagen xerogel skin scaffold named “VitriBand” (VB) behaves like an artificial skin composed of three layers. The uppermost layer consists of an adhesive film dressing, followed by a polyethylene terephthalate film coated with silicone, and the last layer is composed of a collagen xerogel membrane. An in vivo study on mice with full-thickness skin defects compared VB with hydrocolloid dressing and a collagen sponge, showing VB more effectively promoted epithelization and reduced myofibroblast emergence and inflammation than other treatments. This innovation could be a frontline biomaterial for emergency skin injury treatment.^104^ González et al. also created a collagen xerogel cross-linked with colloidal silica particles and oligo urethane, examining its impact on rat skin wound healing. These gels, tested with murine macrophages and human stem cells, demonstrated excellent scaffold characteristics. When composite gel dressings were applied to skin wounds, they exhibited histological characteristics of healed skin that closely mirrored those of intact skin including the epidermis, hair follicles, sebaceous glands, subcutaneous fatty layer, and dermis. These findings highlight collagen-based composite dressings as promising for regenerative skin closure and achieving functional and aesthetic scars.^105^ Table 4 highlights the applications of xerogels in wound healing.

**Table 4:** - Application of Xerogel in Wound Healing

Synthesis method/polymers used	Activities studied	Result	Refs
Alginate grafted with polyethylene glycol methacrylate, cross-linked with strontium ions	Scratch wound assay in HaCaT cells	4.4-fold increase in tensile strength; 55 ± 3.18% Sr^2+^ release by 72 h; 30 ± 4.3% wound closure in 4 h, complete closure by 24 h	^110^
	Collagen synthesis- Sirius red assay in L929 cells		
	Hemostatic activity		
Quercetin borate nanoparticles as a cross-linking agent for Polyvinyl Alcohol via the electrospray technique	Antioxidant activity	Excellent bacteriostasis, antioxidation, self-healing, and accelerated skin regeneration	^65^
	Antibacterial effect		
	In vivo wound healing study using a whole cortex injury model in mice		
Xerogel is composed Poly-lipoic acid (PLA), prepared by the self-polymerization of Lipoic Acid (LA).	Antibacterial activity	99.9% *E. coli* and 99.85% S. aureus killing in 15 min under 808 nm NIR; complete wound repair in 10 days in vivo	^85^
	In vivo full-thickness wound on a rat		
Alginate-based material (ADPM2S) for codelivery of simvastatin and strontium ions	In vitro diabetic model using fibroblast cells (L929), keratinocytes (HaCaT), and macrophages (RAW 264.7)	55% Sr ion and 73% simvastatin release at 24 h; 67% wound closure on L929 cells and 45% on HaCaT cells in 8 h	^79^
	In vitro scratch wound assay using L929 fibroblast cells and HaCaT keratinocyte cells.		
	Collagen synthesis study using L929 fibroblast cells		
	Anti-inflammatory effect		
	Macrophage polarization		
	Gene expression study.		
Alginate conjugated with diamine PEG, grafted with poly(PEGMA), cross-linked with strontium	In vitro scratch wound assay using L929 fibroblast cells	1500% swelling, 400 KPa tensile strength, 78% porosity; 57% GO and 63% POD enzyme release by 24 h	^81^
	Collagen synthesis study using L929 fibroblast cells		
	In vitro ROS detection study using L929 cells		
Sol–gel magnetite matrix with four drugs: chlorhexidine, lidocaine, prednisolone, and chymotrypsin	In vivo excisional full-thickness wound model	∼1.5-fold increase in wound healing rate (21 vs 14 days); strong scar size decrease. The	^86^
″Imitative″ click chemistry based on lipoic acid with disulfide and thioether cross-linking	Antibacterial study	>60% bacteria capture (S. aureus); good ductility and self-healing performance	^87^
Xerogel polymers (3-mercaptopropyltrimethoxysilane (MPTMS) and methyltrimethoxysilane (MTMOS)) and silica nanoparticles with diazeniumdiolate NO-donors	Antimicrobial study	Up to 1.31 μmol NO/mg storage; 2-week NO generation; reduced platelet and bacterial adhesion	^111^
Sol–gel process for mesoporous copper-doped silica xerogels using Tetraethoxy orthosilicate (TEOS) and copper sulfate pentahydrate (CuSO4·5H2O)	Antibacterial study	463.1 m^2^g^–1^ surface area; 99% antibacterial rate against *E. coli* and S. aureus	^90^
Collagen xerogel membrane (dried collagen vitrigel) with adhesive film and silicone coating	Full-thickness wound in vivo study	Promoted epithelialization while inhibiting myofibroblasts and inflammation	^104^
Free radical polymerization of sulfobetaine with oxidative self-cross-linking of reduced keratin	Antioxidant activity, antibacterial activity, and full-thickness in vivo study	Triple-responsive release (acidity, GSH, trypsin); promotes collagen deposition and enhances wound healing, can be ground to powders and reformed in situ	^66^
β-Cyclodextrin conjugated to PEI, cross-linked with epichlorohydrin in the presence of silk fibroin	In vivo study on pressure sore	Better sore-healing efficacy than commercial products; reduced epidermal hyperplasia and neutrophils	^112^
Chitosan/gelatin/polyvinyl alcohol with Thymus pubescens essential oil by freeze-drying	Antimicrobial activity, antioxidant activity, and antibiofilm activity	Excellent antimicrobial efficacy; ∼80% reduction in C. albicans biofilm; 200–700% swelling capacity	^72^
Trilayered asymmetric scaffold: electrospun chitosan-PVA layer + xerogel layer + fibrin glue	Antibacterial activity, In vitro scratch wound assay	Complete bacterial inhibition, significant cell proliferation and migration, and complete wound closure in vitro	^71^
Chitosan-stabilized gold nanoparticles immobilized on SiO_2_/TiO_2_ magnetic xerogel	Antimicrobial assay	Inhibitory effect against *E. coli*; maintained antibacterial activity after magnetic recovery and reuse	^91^
Silk fibroin with riboflavin photosensitizer, free radical cross-linking under UV light	In vitro coagulation index test, external clotting time, rabbit ear artery hemostatic test	90 times water absorption; good hemostatic properties in vitro and in vivo	^63^
Liquid-infused microstructured bioadhesive xerogel formed by covalently cross-linked polyacrylamide	In vitro adhesion test, swelling test, cytocompatibility test, and biodegradation test	Rapid blood absorption and clotting promotion; tough adhesion without compression	^69^
(PAAm) and physically cross-linked chitosan, using freeze-drying.			
Modified sol–gel with PEG molecular imprinting for macroporous chitosan-coated mesoporous silica beads	In vitro plasma coagulation assay, in vitro cytotoxicity test, in vivo hemostasis test	Significantly accelerated coagulation cascade; no exothermic reaction or thermal injury	^96^
Ionotropic cross-linking using Chitosan, tetraethyl orthosilicate (TEOS), gelatin, Sodium Tripolyphosphate, silica nanoparticle, and calcium	In vivo blood clotting efficiency in a rat model with lethal femoral injury	86.7% porosity; > 16-fold improved blood clotting vs commercial products; 2.5 min hemostasis time	^42,68^
UV-assisted method	Water absorption capacity, in vitro and in vivo Hemostasis Test	Enhanced water absorption capacity, along with a good hemostatic effect	^98^
Highly Absorbent Silk Fibroin Protein Xerogel			
Xerogel film composed of Chitosan and gallic acid grated Gelatin prepared using Deep Eutectic Solvent (DES)-assisted extraction and film-casting method;	Antioxidant activity (DPPH assay), Antimicrobial	Strong antioxidant effect (90.6% inhibition at 32 μg/mL), significant antimicrobial activity (zones 17–20 mm), excellent cytocompatibility (>95% cell viability)	^113^
Xerogel film composed chitosan, gelatin, and PVA, fabricated via film-casting method	Antifungal activity (MIC, MFC, agar well diffusion against Candida spp.), antibiofilm activity (XTT assay), cytotoxicity (MTT assay on NIH-3T3 cells), hemocompatibility (RBC lysis test), physicochemical and mechanical characterization.	MIC of 2–8 μL/mL and a minimum fungicidal concentration (MFC) of ≤ 8 μL/mL against Candida species, achieving about 85% biofilm inhibition and nearly 100% fungal colony reduction	^114^

## 5 Emerging Patents on Xerogels

Much research has been conducted on xerogels and their applications in wound healing; however, these studies have not yet been clinically approved. Patents related to xerogels for wound healing focus on developing materials that can effectively promote tissue regeneration and control bleeding.^106^ These xerogels are designed to be highly porous, allowing for better absorption and interaction with biological tissues.^107^ They often incorporate hydrophilic polymers, water-soluble medicaments, and other additives to enhance their healing properties.^106^ Some patents also highlight using xerogels in wound dressings and methods for producing these materials, aiming to improve their mechanical strength,^108^ biocompatibility, and antibacterial properties.^109^ Table 5 highlights the patents related to xerogel’s production method and its application in wound healing.

**Table 5:** - Recent Patents on Xerogels, from the Method of Preparation to Advanced Dressings

Patent number	Title	Description	Refs
US5565142A	Preparation of high porosity xerogels by chemical surface modification.	Prepared the high porosity xerogels by chemical surface modification, followed by drying at vacuum-to-below supercritical pressures	^115^
US5647962A	Process for the Preparation of Xerogels	Modified SiO2 gels (xerogels) are prepared by acidifying an aqueous water glass solution, polycondensing silicic acid, and removing water using an organic solvent	^108^
USOO5738860A	Nonfibrous porous material wound dressing and method of making the material	They prepared a wound dressing composed of porous material, primarily of hydrophilic polymers and water-soluble medicaments, featuring vertically elongated pores formed by leaf-like structures.	^106^
US007 115792B2	Scar-reducing plaster	It consists of scar-reducing plaster with a breathable polyurethane xerogel matrix layer that coats an air- and water vapor-pervious backing film. The plaster is designed with a central scar contact and edge region to minimize peeling during everyday use.	^116^
US8703208B2	Nanometer Mesoporous Silica-Based Xerogel Styptic	Xerogel with good elastic and mechanical properties, formed by adsorbing a large amount of water, promotes wound healing	^107^
US008981139B2	Tertiary-nitrosothol-modified nitric oxide-releasing xerogels and methods of using the same	This invention introduces novel tertiary alkyl thiol and nitrosothiol compounds, as well as methods for creating nitric oxide (NO)-releasing xerogel coatings. The process involves co-condensing a sol precursor solution, coating a substrate, drying to form the xerogel, and treating it with a nitrosating agent.	^117^
US20190388580A1	A superabsorbent polymer hydrogel xerogel sponge, preparation method, and application thereof	The patent describes a superabsorbent polymer hydrogel xerogel sponge with a chitosan skeleton for emergency hemostasis of extensive arteriovenous hemorrhage. It is easy to prepare and offers excellent hemostatic properties, safety, and significant potential in both medical and industrial applications.	^118^
US010736786B2	Hemostatic paste and methods of making thereof	The invention pertains to a flowable hemostatic paste made from cross-linked carboxymethyl cellulose and nontoxic dispersants. Specifically, it involves citric acid cross-linked CMC suspended in a glycerol-containing hygroscopic dispersant and alcohol-functionalized dispersants, such as propylene glycol or 1,3-butanediol.	^109^
US011672864B2	Nanostructured gels capable of controlled release of encapsulated agents	The invention describes self-assembled gel compositions comprising low-molecular-weight gelators, including enzyme-cleavable types. These gels can encapsulate agents for drug delivery, and methods for making and using these compositions are provided.	^119^

## 6 Challenges and Future Outcomes

In the past few years, xerogels have emerged as promising materials for wound dressing applications, offering unique advantages such as enhanced bioinertness, mechanical durability, and the ability to maintain a balanced wound environment to promote healing. However, their widespread adoption in clinical settings faces several challenges that must be addressed. One significant obstacle is the complexity of the production process. Developing xerogels with consistent quality and properties requires precise control over the temperature, pH, and reactant concentrations. For instance, synthesizing chitosan-based xerogels hinges on maintaining a precise degree of deacetylation, which significantly influences their porosity and mechanical properties. Variability in this process can result in inconsistent performance across batches, complicating large-scale manufacturing. Biodegradability and environmental concerns also play crucial roles in the acceptance of xerogels. While many xerogels exhibit promising biocompatibility, their biodegradability can be a concern, and the ecological impact of the synthetic components used in their formulation is a growing concern. Therefore, there is a pressing need for the development of ecofriendly alternatives combining biodegradability with mechanical resilience. Apart from this, the high cost of raw materials such as biopolymers and cross-linking agents, along with the need for complex manufacturing processes like sol–gel synthesis and controlled drying, makes production expensive and technically demanding due to the involvement of high-cost equipment and trained manpower. Regulatory hurdles represent another barrier to the introduction of xerogels to the market. The pathway for approval of medical devices and materials is rigorous, requiring extensive documentation and testing to ensure compliance with the safety and efficacy standards set by regulatory agencies. For example, a company developing a new chitosan xerogel may experience delays in obtaining FDA approval due to the extensive preclinical and clinical data required. Additionally, it is a challenging balancing act to ensure that xerogels possess adequate mechanical strength and resilience for clinical applications while retaining their favorable wound-healing properties. For instance, a gelatin-based xerogel may demonstrate excellent moisture absorption, yet its fragility can limit its utility in high-mobility areas, where structural integrity is essential. Optimizing cross-linking density and incorporating reinforcing nanomaterials are current strategies being explored to overcome this limitation. Furthermore, despite promising findings in laboratory settings, the limited availability of comprehensive clinical trials hampers understanding of xerogels’ long-term effects and effectiveness in real-world applications. Despite promising in vitro and in vivo results, a paucity of comprehensive clinical trials to evaluate xerogels in real-world wound care scenarios. Without such data, it is difficult to establish long-term efficacy, safety, and cost-benefit ratios. More extensive studies are needed to validate their benefits and assess the potential risks associated with their use. Addressing these multifaceted challenges will be crucial for unlocking the full potential of xerogels as advanced wound dressing materials. Collaborative efforts among researchers, industry stakeholders, and regulatory bodies are essential to overcoming these hurdles and bringing innovative solutions to market. Ultimately, the future of biopolymeric xerogels depends on a concerted effort across academia, industry, and regulation to ensure that innovations not only advance scientific understanding but also deliver safe, effective, and accessible healthcare solutions.

## 7 Conclusions

A comprehensive understanding of the wound healing process critically involves infection control, effective exudate management, maintaining a supportive healing environment, and ensuring adequate nutrition and hydration. Recent advancements in wound care materials have introduced innovative solutions, among which xerogels stand out for their unique properties and broad potential applications. Xerogels have a high porosity and a large surface area, which enables them to absorb wound exudates efficiently while promoting tissue regeneration. It also provides a moist wound environment, which is critical for optimal healing. Apart from that, xerogels enhance essential cellular processes such as attachment, proliferation, and differentiation. These materials can be embedded with antibacterial agents, growth factors, and therapeutic actives, thereby improving their effectiveness in accelerating healing and preventing infections. The rise in xerogel-related patents reflects ongoing innovation in wound care. These focus on integrating hydrophilic polymers, therapeutic agents, and advanced fabrication methods to enhance strength, biocompatibility, and porosity. Continued research and development (R&D) is key to optimizing formulations and expanding clinical use. With their combined properties of hemostasis, enhanced tissue regeneration, and the ability to carry a diverse range of therapeutic agents, xerogels represent a significant step forward in wound healing technology. In conclusion, xerogels are poised to play a vital role in the future of wound care, offering innovative, adaptable, and effective solutions to meet the evolving needs of patients and healthcare providers alike.

## Data Availability

No data were used for the research described in the article.

## Glossary

BCI: blood clotting index
CMC: carboxymethyl cellulose
CSSX: chitosan-containing mesoporous silica
ECM: extracellular matrix
FDA: food and drug administration
FTIR: Fourier transform infrared
GO-POD: glucose-peroxidase
IUPAC: International Union of Pure and Applied Chemistry
LIMBs: liquid-infused microstructured bio-adhesives
m-SXCu: mesoporous copper-doped silica xerogels
NIR: near-infrared
NO: nitric oxide
PCL: poly-caprolactone
PDT: photodynamic therapy
PEG: polyethylene glycol
PVA: polyvinyl alcohol
ROS: reactive oxygen species
SIM: simvastatin
SPA: sodium polyacrylate
SiNPs: silicon dioxide nanoparticles
St: strontium
VB: VitriBand
